# Associations between person-environment fit and mental health - results from the population-based LIFE-Adult-Study

**DOI:** 10.1186/s12889-024-19599-z

**Published:** 2024-08-01

**Authors:** Franziska U. Jung, Margrit Löbner, Francisca-Saveria Rodriguez, Christoph Engel, Toralf Kirsten, Nigar Reyes, Heide Glaesmer, Andreas Hinz, A. Veronica Witte, Hannes Zacher, Markus Loeffler, Arno Villringer, Melanie Luppa, Steffi G. Riedel-Heller

**Affiliations:** 1 Institute of Social Medicine, Occupational Health and Public Health (ISAP), Leipzig University, Ph.-Rosenthal-Str. 55, Leipzig, 04103 Germany; 2 German Center for Neurodegenerative Diseases (DZNE), RG Psychosocial Epidemiology & Public Health, Greifswald, Germany; 3 Institute for Medical Informatics, Statistics and Epidemiology, Leipzig University, Leipzig, Germany; 4 LIFE- Leipzig Research Centre for Civilization Diseases, Leipzig University, Leipzig, Germany; 5 Medical Informatics Center, Department of Medical Data Science, Leipzig University Medical Center, Leipzig, Germany; 6 Department of Medical Psychology and Medical Sociology, Leipzig University, Leipzig, Germany; 7 Cognitive Neurology, University of Leipzig Medical Center, Leipzig, Germany; 8 Department of Neurology, Max Planck Institute for Human Cognitive and Brain Sciences, Leipzig, Germany; 9 Wilhelm Wundt Institute of Psychology, Leipzig University, Leipzig, Germany; 10 Clinic of Cognitive Neurology, University of Leipzig Medical Center, Leipzig, Germany

**Keywords:** Person organization fit, Demands abilities fit, Needs supplies fit, Depression, Anxiety

## Abstract

Within occupational settings, mental health of employees can be affected by complex interactions between individuals and their work environment. The aim of this cross-sectional analysis was to investigate the association between person-environment fit and mental health in employees. Data of *n* = 568 participants from the LIFE adult cohort study was analysed, including socio-demographic characteristics, three dimensions of person-environment fit (P-E fit), symptoms of depression and anxiety. Assessment took place between 2017 and 2021. Statistical analysis included descriptive analyses as well as generalized linear regression models adjusted for age, gender, socioeconomic status, marital status, and job status. Correlational analysis revealed significant associations between age, marital status, SES, employment status, symptoms of depression and anxiety and P-E fit. According to regression models, greater perceived fit between person and organization was associated with lower depression scores and lower symptoms of anxiety. Higher perceived fit between demands and abilities was significantly related to lower severity of depression and anxiety. Similarly, participants reporting a higher fit between needs and supplies, exhibited less symptom severity regarding depression and anxiety. These results underline the importance of person-environment fit regarding mental health. Finding ways to obtain an optimal balance should not only be recognized as an important factor for health and well-being, but might also be beneficial for organizations and employers in the long-term.

**Supplementary Information:**

The online version contains supplementary material available at 10.1186/s12889-024-19599-z.

## Introduction

### Changing work arrangements and the concept of occupational fit

Our working world is undergoing rapid change. Advancing digitalization is not only changing job profiles; working conditions and requirements have also significantly changed. This may require a great willingness to change, learn, or adapt on the part of employees. A misfit between work-related requirements and personal abilities and qualifications may result in negative work experiences as well as health-related consequences, aligning with assumptions described by the demand control model (later known as the demand control support model) [1, 2]. According to this model, job demands are handled or compensated by putting in greater cognitive, emotional, or professional efforts, which may lead to physical or mental stress, especially if job demands exceed the employee’s abilities. In addition, job resources, such as training opportunities or feedback, may help to counteract fatigue and exhaustion caused by job demands. In this context, the fit between person and work environment is of great relevance in order to achieve sustained employability and overall health.

The concept of „fit“ (or congruence) within an occupational context has often been focused on by work and organizational psychologists [3–5]. In this context, fit with one’s job, work team, or organization/company, also described as person-environment-fit (P-Efit), has been shown to be crucial exploring employee and employer overlapping interests regarding career decision-making and staffing. Job applicants may choose organizations or jobs based on their perceived fit regarding their own aptitude or organizational norms they can identify with. Further, organizations may want to attract future employees that are eligible for the position they apply for in order to reduce staff fluctuation or turnover and therefore be cost-effective [6]. Based on a comprehensive meta-analysis by Kristof-Brown et al. [7], it is important to include different types of fit regarding work-related attitudes, behaviors and consequences. According to Cable and DeRue [8], employees differentiate between three types of person-environment fit: person-organization fit, needs-supplies fit, and demands-abilities fit. According to the authors, person-organization fit is related to organization-focused outcomes, such as citizenship behavior or organizational identification. The second dimension, needs-supplies fit, is rather related to outcomes associated with the job itself or career (e.g., job and career satisfaction). Third, the extent to which abilities and skills match the demands of a certain job is reflected by demands-abilities fit perceptions. Regarding organizational consequences, perceived over-qualification and demands-abilities misfit has been shown to be significantly related to counterproductive work behavior [9] and increased turnover intentions [10, 11]. Similarly, better person-environment fit have been shown to be associated with higher life and occupational satisfaction [12]. However, it has been shown that sociodemographic or professionally relevant factors may lead to mixed results when investigating P-E fit. For example, the association between person-organization fit and job satisfaction may be more relevant to older employees [13]. Therefore, it is important to take into account sociodemographic and occupational characteristics.

### Person-environment fit and health

Although the epidemiological numbers of mental illnesses in the general population as such do not increase, and rather remain constant at around 30% [14], studies also found a general rise in diagnosis or incidence frequency, as well as disability-adjusted life-years [15, 16].

According to the WHO guidelines on mental health at work, the workplace is an important setting for protecting and promoting psychological wellbeing [17]. In fact, the three dimensions of person-environment fit described above may act as a contributing factor, being associated with mental health. In this context, it has been found that personal-job fit (or misfit) may directly and indirectly impact mental health, mediated by burnout and emotional labor [18]. Additionally, research has shown that misfits between employees and their organization, as well as between individual needs and environmental supplies can also result in more mental and physical symptoms of exhaustion and a greater severity of burnout [19–21]. A previously published study further investigated the link between person-job fit, overall health, and depression [22]. Here, need-supply fit was significantly associated with health status and depressive symptoms. However, the focus was on domestic workers only, and due to the characteristics of these professions, only two dimensions (demand-ability fit and need-supply fit) were included in the analyses.

Therefore, previous findings on associations between P-E fit and mental health are rather limited and may not reflect all facets of this research area. Especially the person-perspective has often been left out of theoretical models, that rely on traditional work arrangements. Based on the concept of person-environment fit by Cable and DeRue [8] mentioned before, the aim of the current study was to fill this gap and explanatorily investigate the link between subjective fit perception (P-E fit) and mental health (symptoms of depression and anxiety) in a heterogeneous sample of employees. Therefore, the first aim was to investigate whether the three dimensions of P-E fit are associated with sociodemographic and occupational characteristics, as well as symptoms of depression and anxiety. In a second step, regression analysis was conducted in order to investigate whether employees’ perception of fit between person and organization (Model 1), demands and abilities (Model 2), and needs and supplies regarding the workplace (Model 3), may be associated with greater symptoms of depression and/or anxiety. Here, sociodemographic and occupational characteristics were considered as potential confounding variables.

## Method

### Data collection and recruitment

Data were taken from the first follow-up wave of the LIFE-Adult-Cohort of the Leipzig Research Centre for Civilization Diseases, and includes a population-based sample of people living in Leipzig (Eastern Germany). The sample was randomly drawn from local resident’s registration office (Baseline: between 2011 and 2014) and consists of individuals aged between 18 and 80 [23]. The first follow-up assessment took place between 2017 and 2021. Overall, participants received an assessment battery at the study centre, for example with regard to sociodemographic information, medical history, details on lifestyle factors, as well as medical examinations. The sole conditions for exclusion were pregnancy and a lack of proficiency in German. Further information on the study procedure as well as data collection and ethical considerations have been described elsewhere in greater detail [23, 24].

The instrument measuring subjective P-E fit was only included within follow-up assessments that took place at the study centre. Overall, *n* = 1,703 participants filled in the P-E fit questionnaire. Furthermore, the sample was restricted to people that were currently employed (*n* = 1,135 were excluded^1^). Therefore, the study sample that was used for the current analysis consisted of 568 participants. There was no significant difference between the participants who were included versus those who were excluded regarding gender (*p* = .071). However compared to those that were excluded, the sample under investigation was significantly higher educated (*p* < .001) and had a higher socioeconomic status (*p* < .001).

The prevalence of missing values in this sample was below 5%, therefore no procedure was applied, as suggested in the literature [25].

### Sociodemographic characteristics

#### Instruments

The independent variable, P-E fit, was measured using the 9-item perceived fit scale by Cable and DeRue, which has been shown to have good psychometric properties [8]. The scale is subdivided into three dimensions each containing three items: person-organization fit (α = 0.875), needs-supply fit (α = 0.823) and demands-abilities fit (α = 0.924). Example items can be found in the appendix. All items were answered using a 5-point scale (1 = not at all; 5 = entirely). Higher means scores indicate greater subjective fit perceptions across all three dimensions. The three-factor structure of the scale has been shown to be the most reliable tool fro global assessment of person-environment fit with good psychometric properties [8, 26, 27]. The German translation has been used in similar settings before [28].

The first outcome variable with regard tosymptoms of depression, has been assessed using the Center for Epidemiologic Studies Depression Scale (CES-D, [29]), including 20 items on symptoms such as depressed mood or hopelessness during the last week, using a 4-point-scale (0 = never/almost none of the time, 3 = most/ all of the time). The German version of this self-report scale has been shown to have good psychometric properties [30]. Higher sum scores indicate greater severity of symptoms (range in this sample: 0–47). Cronbach’s alpha in this sample was 0.850.

The second parameter of mental health, symptoms of anxiety, has been assessed using the Generalized Anxiety Disorder scale (GAD-7, [31, 32]), which measures symptoms of anxiety with seven items. Again, this instrument has been widely used, showing good psychometric properties. Higher sum scores, indicate greater severity of symptoms (range in this sample: 0–19). Cronbach’s alpha in this sample was 0.863.

As confounding variables, socioeconomic status (SES) of the participants in this study was calculated based on already established standards and included information on education, occupation, and income [33]. The SES is subdivided into low, middle and high. Further information on how this index is generated, can be found elsewhere [33].

### Data analysis

The analyses were based on cross-sectional data. Descriptive statistics are shown as means with standard deviations or number of cases with percentages. As a first step, correlations between each dimension and the covariates were conducted in order to detect any associations between variables *(*categorical: Kruskal Wallis H, continuous: Spearman).

Associations between P-E fit, symptoms of depression, and anxiety were then examined using multivariate regression analyses. Two models were evaluated, distinguishing between the three P-E fit subscales as dependent variables. Due to the skewed distribution of the variables under investigation, generalized linear regression models (GLM) have been applied (using gamma distribution and a log link function).

All models were adjusted for age, gender, socioeconomic status (SES), marital status, and job status. In addition, data were weighed for age, gender and level of education to be representative of the German general population in 2016. The software program StataSE Version 16 has been used for all statistical analyses. Statistical significance was defined with *p* < .05.

## Results

Sociodemographic information regarding the study sample can be found in Table 1. The mean age of the participants eligible for analysis was 50.5 (SD = 10.7) and the majority were male (57.0%) and currently employed by contract (78.9%) at the time of the data collection.

Overall, mean scores reflecting the severity of depression were 8.5, and 3.3 regarding symptoms of anxiety.

**Table 1:** Sociodemographic characteristics of the study sample

	Study Sample (*n* = 568)
Age, M (SD) range: 26–77 years	50.5 (10.7)
Gender (n, %)	
female	244 (43.0)
male	324 (57.0)
Marital Status, n (%)	
married	315 (55.5)
single	188 (33.1)
divorced	58 (10.2)
widowed	5 (0.9)
missing	2 (0.4)
Socioeconomic Status, n (%)	
low	52 (9.2)
middle	342 (60.2)
high	172 (30.3)
missing	2 (0.4)
Employment status, n (%)	
self-employed	100 (17.6)
employed	448 (78.9)
missing	20 (3.5)
Depression (CES-D), M (SD) range: 0–47	8.5 (6.7)
Anxiety (GAD), M (SD) range: 0–19	3.3 (3.3)
Person-Environment Fit, M (SD) range 1–5	
person-organization Fit	3.5 (0.9)
demands-abilities Fit	4.1 (0.8)
needs-supplies Fit	3.7 (1.0)

Note: M = mean; SD = standard deviation; values do not add to 100% because of rounding

**Table 2:** Associations between the three dimensions of person-environment fit and participants characteristics

	Person-Environment Fit		
	Person-Organization Fit M (SD)	Demands-Abilities Fit M (SD)	Needs-Supplies Fit M (SD)
**Age** (yrs)	*p* < .001	*p* = .009	*p* = .032
21–40	3.2 (0.9)	3.9 (0.8)	3.5 (1.1)
41–60	3.5 (0.9)	4.1 (0.7)	3.7 (1.0)
> 61	3.9 (0.9)	4.2 (0.8)	3.9 (1.1)
**Gender**	*p* = .365	*p* = .991	*p* = .735
female	3.5 (1.0)	4.1 (1.0)	3.7 (1.0)
male	3.6 (0.9)	4.1 (0.8)	3.7 (1.0)
**Marital Status**	*p* = .006	*p* = .003	*p* = .004
married	3.6 (0.9)	4.2 (0.7)	3.8 (1.0)
single	3.4 (0.9)	4.0 (0.8)	3.5 (1.0)
divorced	3.5 (1.1)	4.3 (0.7)	3.7 (1.1)
widowed	3.4 (1.1)	4.3 (1.5)	3.9 (1.7)
**SES**	*p* = .006	*p* = .020	*p* = .001
low	3.3 (0.9)	3.8 (1.0)	3.2 (1.2)
middle	3.5 (0.9)	4.1 (0.8)	3.7 (1.0)
high	3.7 (0.9)	4.3 (0.7)	3.9 (0.9)
**Employment status**	*p* = .001	*p* = .004	*p* < .001
self-employed	4.1 (0.8)	4.3 (0.9)	4.1 (0.9)
employed	3.4 (0.9)	4.1 (0.8)	3.7 (1.0)

Note: M = mean, SD = standard deviation; Kruskal Wallis H was used to test for significant associations between variables

Associations between the three dimensions of the outcome variable P-E fit (person-organization fit, demands-abilities fit and needs-supplies fit) with sociodemographic characteristics, employment status, depression and anxiety are summarized in Tables 2 and 3. Overall, no significant association was found between gender and all three dimensions. Age (in categories), marital status, socioeconomic status and employment status were significantly related to all three concepts of person-environment fit. In other words, higher age, higher SES and being self-employed was rather associated with better fit across all three dimensions. With regard to marital status, being married was associated with higher person-oragnaization fit, whereas being widowed was associated with higher demands-abilities, as well as needs-supplies fit (see Table 2).

**Table 3:** Associations between the three dimensions of person-evironment fit and mental health

	Person-Evironment Fit		
	Person-Organization Fit	Demands-Abilities Fit	Needs-Supplies Fit
**Depression (CES-D)**	Spearman’s ρ=-0.019, *p* < .001	Spearman’s ρ=-0.252, *p* < .001	Spearman’s ρ =-0.259, *p* < .001
**Anxiety (GAD-7)**	Spearman’s ρ=-0.149, *p* = .001	Spearman’s ρ=-0.167, *p* < .001	Spearman’s ρ=-0.164, *p* < .001

Note: CES-D = Center for Epidemiologic Studies Depression Scale, GAD-7 = Generalized Anxiety Disorder, Spearman was used for statistical analysis of associations between variables

**Fig. 1:**
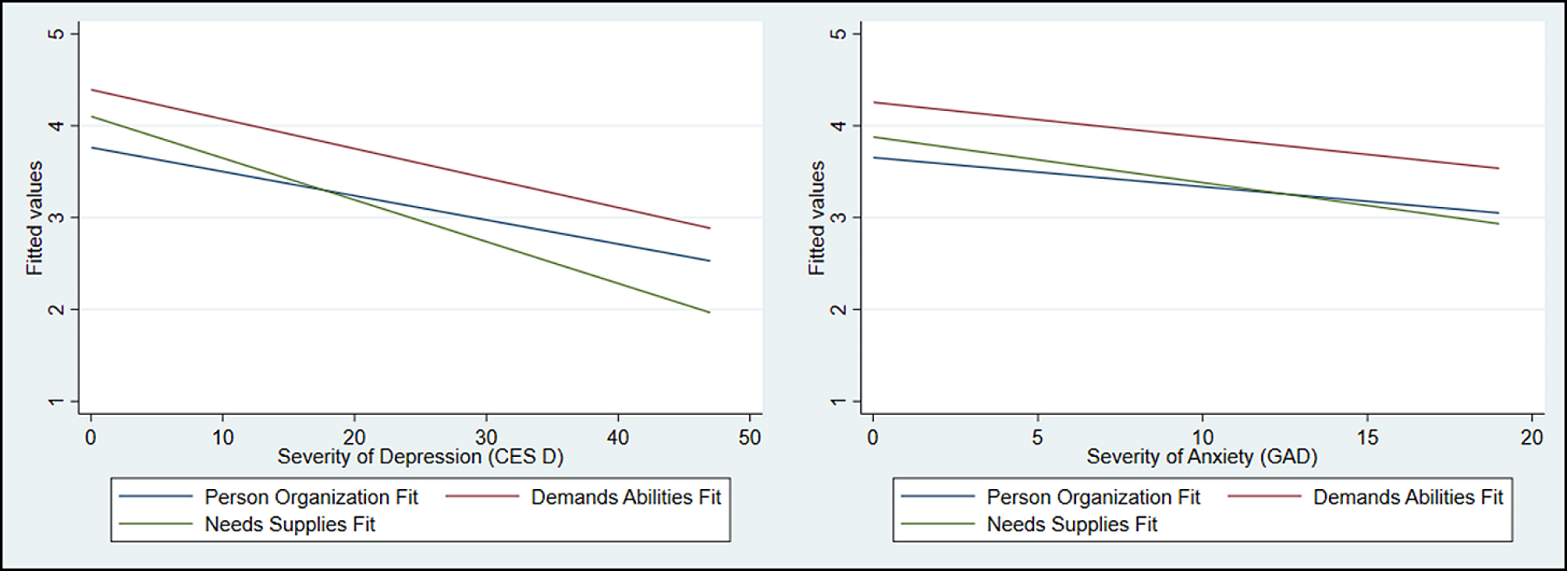
Association between the three concepts of fit and severity of both depression and anxiety Note: CES-D = Center for Epidemiologic Studies Depression Scale; GAD = Generalized Anxiety Disorder

Symptoms of depression were negatively associated with all three dimensions of P-E fit (Table 3; Fig. 1). In other words, greater symptoms of depression were associated with lower scores regarding the fit. Symptoms of anxiety were also significantly related to all three concepts of fit, meaning that higher scores regarding anxiety were related to lower scores regarding the fit (Table 3; Fig. 1).

Furthermore, regression analyses confirmed our hypotheses and revealed a significant association between all three concepts of person-job fit and depression as well as anxiety (Tables 4 and 5), when controlling for gender, age, socioeconomic status, marital status and employment status. In other words, greater subjectively perceived fit between the person and the organization was associated with lower scores regarding symptoms of depression (ß = -0.185, *p* < .001) and symptoms of anxiety disorder (ß = -0.135, *p* = .019). Higher perceived fit between demands and abilities was also significantly related to lower severity of depression (ß = -0.307, *p* < .001) and anxiety (ß = -0.207, *p* = .006). Similarly, participants reporting a higher perceived fit between needs and supplies, exhibited less symptom severity regarding depression (ß = -0.247, *p* < .001) and anxiety (ß = -0.189, *p* = .003).

**Table 4:** Regression models with severity of depression as the outcome variable and each dimension of person-environment fit as the independent variable (model 1: person-organization (P-O fit); model 2: demands-abilities (D-A fit), model 3: needs-supplies (N-S fit))

	Outcome variable: Depression					
	Model 1: P-O Fit (*n* = 532)		Model 2: D-A Fit (*n* = 518)		Model 3: N-S Fit (*n* = 518)	
	β	95% Conf. Interval	β	95% Conf. Interval	β	95% Conf. Interval
**Independent Variable**	− 0.185***	**− 0.268;-0.103**	− 0.307***	**− 0.414;-0.199**	− 0.247***	**− 0.330;-0.165**
**Gender** (ref. male)	0.074	− 0.080;-0.228	0.044	− 0.113;-0.201	0.077	− 0.074;-0.228
**Age**	0.002	− 0.006;-0.010	0.002	− 0.006;-0.010	0.002	− 0.006;-0.010
**Socioeconomic status** *(ref. low)*						
middle	− 0.182	− 0.515;-0.151	− 0.177	− 0.553;-0.199	− 0.131	− 0.436;-0.174
high	− 0.385*	− 0.726;-0.045	− 0.356	− 0.740;-0.028	− 0.290	− 0.609;-0.028
**Marital status (** *ref. married* **)**						
single	0.118	− 0.047;- 0.284	0.091	− 0.072;-0.255	0.064	− 0.105;-0.232
divorced	0.123	− 0.085;- 0.332	0.180	0.028; 0.388	0.085	− 0.103;-0.272
widowed	0.166	− 0.473;-0.806	− 0.515	-1.214;-0.215	− 0.503	-1.219;-0.213
**Employment status** *(ref. self-employed)*	− 0.111	− 0.303; 0.081	− 0.082	− 0.268;-0.104	− 0.143	− 0.338;- 0.053
**constant**	2.917***	2.233;-3.601	3.506***	2.825;-4.187	3.151***	2.532;-3.770
**Correlation coeff.**	0.248		0.289		0.329	
**R** ^**2**^	0.061		0.083		0.108	

Weighed for age, gender and level of education to be representative of the German general population in 2016; *: *p* < .05, **: *p* < .01, ***: *p* < .001

**Table 5:** Regression models with severity of anxiety as the outcome variable and each dimension of person-environment fit as the independent variable (model 1: person-organization (P-O fit); model 2: demands-abilities (D-A fit), model 3: needs-supplies (N-S fit))^1^

	Outcome variable: Anxiety					
	Model 1: P-O Fit (*n* = 464)		Model 2: D-A Fit (*n* = 451)		Model 3: N-S Fit (*n* = 451)	
	β	95% Conf. Interval	β	95% Conf. Interval	β	95% Conf. Interval
**Independent Variable**	− 0.135*	**− 0.24;-0.023**	− 0.207*	**− 0.355;-0.058**	− 0.189**	**− 0.313;-0.065**
**Gender** (ref. male)	0.279*	0.064;0.495	0.274*	0.053;0.496	0.293**	0.077;0.509
**Age**	− 0.007	− 0.019;0.005	− 0.008	− 0.020;0.004	− 0.006	− 0.018;0.006
**Socioeconomic status** *(ref. low)*						
middle	− 0.283	− 0.860;0.293	− 0.312	− 0.929;0.305	− 0.230	− 0.789;0.329
high	− 0.330	− 0.908;0.248	− 0.340	− 0.955;0.275	− 0.241	− 0.804;0.322
**Marital status** *(ref. married)*						
single	0.072	− 0.153;0.297	0.049	− 0.175;0.273	0.041	− 0.188;0.269
divorced	0.213	− 0.087;0.512	0.253	− 0.043;0.550	0.205	− 0.093;0.503
widowed	− 0.329	− 0.743;0.086	− 0.462**	− 0.807;-0.118	− 0.497**	− 0.846;-0.148
**Employment status** (ref. self-employed)	− 0.112	− 0.407;0.182	− 0.091	− 0.382;0.200	− 0.154	− 0.458;0.151
**constant**	2.204***	1.028;3.381	2.645***	1.405;3.885	2.371***	1.243;3.498
**Correlation coeff.**	0.227		0.226		0.240	
**R** ^**2**^	0.052		0.051		0.058	

Weighed for age, gender and level of education to be representative of the German general population in 2016; *: *p* < .05, **: *p* < .01, ***: *p* < .001

## Discussion

The aim of this study was to build on previous research, investigating the consequences of three types of person-environment fit in occupational settings: between person and organization, (individual) demands and abilities, as well as needs and supplies.

The associations between these three dimensions and several sociodemographic, occupational and mental health-related characteristics revealed the following results:

In general, symptoms of depression were associated with all three dimensions, even after adding several confounding variables (age, gender, marital status, socioeconomic status, and employment status) within regression models. In other words, higher fit between person and organization, demands, and abilities, as well as needs and supplies, was significantly related to fewer symptoms of depression.

In addition, symptoms of anxiety were also significantly related to all three concepts of fit, meaning that higher scores regarding fit were related to fewer symptoms of anxiety.

These findings could be explained by theoretical models [1, 2] and are consistent with findings [22, 34]. For instance, when job-related demands are too high or exceed employees‘ abilities, it may result in mental health issues [18]. In fact, poor fit between person and organization has been shown to be a source of chronic psychological distress [34], which could explain the current results regarding the association between P-Efit and mental health. On the other hand, individuals with anxiety or depression disorders may have problems at work and thus perceive P-Efit as being lower. In this context, it has been shown that individuals with high work-anxiety may over-report negative workplace conditions, such as job control [35].

The topic under investigation is of great relevance, especially in the context of fast changing work environments (due to innovations and globalization) and lack of personal in many professions, that include high mental and emotional demands, such as health care [36]. Previous research has demonstrated several negative effects associated with occupational misfit, such as job and life satisfaction [12], employee work attitudes [37], emotional exhaustion, and distress [38]. Only a few studies focused on health, specifically mental health outcomes. However, it could be argued that negative consequences related to satifaction or exhaustion may also explain our findings with regard to depression and anxiety. A very recent study, investigating the link between person-job fit and physical and mental health has demonstrated that both burnout and emotional labor may mediate this association [18]. Unfortunately, only person-job fit was investigated in this study, and only medical staff working in hospitals was recruited for participation. Therefore, the results may not be generalizable to other professions. In line with these results, it has been shown that all three subjective fit perceptions at the workplace are negatively correlated with distress and emotional exhaustion in a sample of young project management professionals [38]. Similar results were obtained from employees working in mental health care settings [39, 40]. Another study showed that demand-ability fit as well as need-supply fit were significantly related to depressive symptoms [22]. These findings were based on a sample containing only domestic workers. Similarly, Park et al. [41] found a significant association between demands-abilities fit and depression, but not between needs-supplies fit and depression. However, the sample size was rather small, including 90 employees working in two different companies. With regard to health-related outcomes, greater fit between person and job, as well as person and organization, may result in higher psychological safety as well as safety behavior among employees [42]. According to the authors, if employees perceive that they fit into the position or workplace well, they may develop a sense of belonging or identity, which may be transferred to organizational commitment or a psychological contract, leading to increased safety behaviors, that are beneficial for their own and their colleagues’ health and safety. The current sample included employees with a variety of occupational backgrounds and hierarchies and is therefore more representative of the general public, showing that person-enironment fit should not be overlooked as an important factor related to employees’ mental health. So far, individual characteristics such as gender, age, or employment status (i.e., employed vs. self-employed) may influence the association between P-E fit and mental health to some extent, but research is still insufficient. According to the current study, gender was only significantly associated with symptoms of anxiety, when person-organization, demands-abilities, as well as needs-supplies fit were included as predictors, as women showed more symptoms of anxiety compared to men. So far, there is only one study that has investigated gender differences with regard to mental health and person-organization fit [34]. Here, higher levels of identification with the organization led to worse mental health status in women, whereas having the same goals and values as the organization was beneficial for men’s mental health. The authors argue that over-identification may lead to negative outcomes related to mental health. Unfortunately, these results were based on data regarding mental health in general, including somatic complaints; therefore, the results cannot be directly compared with the current investigation with a focus on depression and anxiety. Future research could investigate the mediating role of gender in the association between person-environment fit and anxiety in greater detail, as previous studies concentrated on job satisfaction or turnover intention but not mental or psychological health.

### Limitations

The study has some limitations. Perceived fit between person and job, demands, and abilities or needs and supplies, was rated using subjective measures. Individuals may differ in their own perceptions, for example, by overrating or underrating their own abilities. Therefore, it could be interesting to include objective measures as well. The data under investigation is further limited due to its cross-sectional design. Longitudinal studies may be able to determine the direction of causality. Specifically, they could show whether occupational misfit leads to greater symptoms of mental health issues or whether individuals with more symptoms of depression or anxiety have, for example, a poorer person-job fit perception, because they are more self-critical [43] or pessimistically biased regarding their self-evaluation [44]. Future research could also determine (risk) groups depending on symptom load and P-E fit and their differences in relation to sociodemographic or occupational characteristics in order to develop specific interventions that may be implemented at different workplaces. Given its correlation with extended sick leave and work disability, work-anxiety may be viewed as a risk factor for low P-Efit [35]. In addition, no differentiation between occupational sectors has been made, which could influence the results described above. Despite these limitations, this is the first study that sheds light on the importance of all three constructs of fit (person-organization, demands-abilities, and needs-supplies) an their association with mental health in a heterogeneous sample with broad socio-demographic and occupational characteristics.

### Implications and conclusion

Several implications can be derived from our results. First, it was shown that person-environment fit in terms of demands and abilities, needs and supplies, but also between the employee and the job, has an important association with the employee’s well-being and psychological health. Currently, individual needs related to their occupation are becoming more and more important, and employers should find ways to establish individualized support as part of their leadership repertoire. Moreover, culture-change interventions that allow for these individual needs could be developed in order to improve employees’ mental health.

Future research could build up on these findings and analyze longitudinal data, especially in order to investigate long-lasting consequences with regard to employability and (early) retirement. Especially as previous research has demonstrated, person-job as well as person-organization fit may impact job satisfaction and the intention to quit, even if both should be thought of as two separate constructs [45].

Second, it has been shown that person organization fit is not static. It rather develops over time as individuals, as well as organizations influence each other constantly [46]. Therefore, longitudinal designs may be helpful to investigate whether changes in fit may reflect changes in depression or anxiety.

In summary, these results clearly show that there is an association between person-environment fit and mental health, independent of individual characteristics such as age or gender, and across different occupations. Therefore, future studies could concentrate on replicating these results using a longitudinal design to determine the direction of causality, and based on these findings, develop interventions that can be implemented at the workplace in order to preserve employees’ mental health and maintain their workability.

### Electronic supplementary material

Below is the link to the electronic supplementary material.

Supplementary Material 1

## Data Availability

Data cannot be shared openly due to participant privacy but are available on request from the corresponding author (FUJ).

## Footnotes

1 The LIFE study covers a main age range from 40 to 79 years, however, a particular deep phenotyping in participants above the age of 60 should be mentioned, which explains the high number of participants being excluded because of their employment status.

## Declarations

The author(s) acknowledge support from the German Research Foundation (DFG) and Universität Leipzig within the program of Open Access Publishing.
