# Supplementary material for: Associations between person-environment fit and mental health - results from the population-based LIFE-Adult-Study
Source: BMC Public Health. 2024 Aug 1;24:2083. doi: 10.1186/s12889-024-19599-z (PMC11295583; doi:10.1186/s12889-024-19599-z)
Supplement: Supplementary file 1 — Supplementary Material 1 [file 12889_2024_19599_MOESM1_ESM.docx]

**Supplementary Material**

Person-Environment Fit – Item Description

The things that I value in life are very similar to the things that my organization values.

My personal values match my organization's values and culture.

My organization's values and culture provide a good fit with the things that I value in life.

There is a good fit between what my job offers me and what I am looking for in a job.

The attributes that I look for in a job are fulfilled very well by my present job.

The job that I currently hold gives me just about everything that I want from a job.

The match is very good between the demands of my job and my personal skills.

My abilities and training are a good fit with the requirements of my job.

My personal abilities and education provide a good match with the demands that my job places on me.

Note: items were rated on a 5-point scale ranging from 1 (not at all) through 5 (completely)

Reference: Cable, D.M. & DeRue, D.S. (2002). The Convergent and Discriminant Validity of Subjective Fit Perceptions. Journal of Applied Psychology. 87(5). 10.1037/0021-9010.87.5.875
